# Cold Atmospheric Plasma: A Promising Complementary Therapy for Squamous Head and Neck Cancer

**DOI:** 10.1371/journal.pone.0141827

**Published:** 2015-11-20

**Authors:** Christian Welz, Steffen Emmert, Martin Canis, Sven Becker, Philipp Baumeister, Tetsuji Shimizu, Gregor E. Morfill, Uli Harréus, Julia L. Zimmermann

**Affiliations:** 1 Department of Otorhinolaryngology, Head & Neck Surgery, Georg-August-University, Göttingen, Germany; 2 Department of Dermatology, Venereology and Allergology, Georg-August-University, Göttingen, Germany; 3 Department of Otolaryngology, Head and Neck Surgery, Johannes Gutenberg-University, Mainz, Germany; 4 Department of Otorhinolaryngology, Head & Neck Surgery, Ludwig-Maximilians-University, Munich, Germany; 5 Terraplasma GmbH, Garching, Germany; 6 Department of Otolaryngology, Head and Neck Surgery, evangelical hospital Düsseldorf, Germany; Massachusetts General Hospital, UNITED STATES

## Abstract

Head and neck squamous cell cancer (HNSCC) is the 7^th^ most common cancer worldwide. Despite the development of new therapeutic agents such as monoclonal antibodies, prognosis did not change for the last decades. Cold atmospheric plasma (CAP) presents the most promising new technology in cancer treatment. In this study the efficacy of a surface micro discharging (SMD) plasma device against two head and neck cancer cell lines was proved. Effects on the cell viability, DNA fragmentation and apoptosis induction were evaluated with the MTT assay, alkaline microgel electrophoresis (comet assay) and Annexin-V/PI staining. MTT assay revealed that the CAP treatment markedly decreases the cell viability for all tested treatment times (30, 60, 90, 120 and 180 s). IC 50 was reached within maximal 120 seconds of CAP treatment. Comet assay analysis showed a dose dependent high DNA fragmentation being one of the key players in anti-cancer activity of CAP. Annexin-V/PI staining revealed induction of apoptosis in CAP treated HNSCC cell lines but no significant dose dependency was seen. Thus, we confirmed that SMD Plasma technology is definitely a promising new approach on cancer treatment.

## Introduction

Head and neck squamous cell cancer (HNSCC), including malignancies of the oral cavity, the pharynx and the larynx, is the 7^th^ most common cancer worldwide contributing over 4% of the total number of new cancer cases in 2012. This means, nearly 600.000 new diseases are diagnosed per year worldwide [1]. For 2014, 55.070 new cases and 12.000 deaths are estimated in the USA. The average 5-year survival rate is about 50%, which is mainly due to the high rate of metastasis, second malignancies and especially local recurrence of these tumors [2]. Despite new approaches in the treatment of head and neck cancer such as monoclonal antibodies the overall survival could not be essentially increased in the last decades, which asks for the development of novel antitumoral approaches and technologies. Because of its characteristics, cold atmospheric plasma (CAP) is the most promising and potential development in this area.

Plasma can simplified be described as a partially or fully ionized gas. Synthetic generated plasmas like the argon plasma coagulator are already established tools in medicine [3]. However, because of their high temperature (>10.000°C) they can only be used for ablation or coagulation purposes [4]. Since a few years plasmas can be generated with an ion temperature which is close to room temperature. These so called cold atmospheric plasmas have already proved their immense anti-microbial efficacy in clinical trials and opened up a wide range for medical applications especially for all heat-sensitive and vital surfaces such as human skin or mucosa [5–10].

Besides the antimicrobiotic application, recent results raised an antitumoral potential of CAP. The biological effects and mechanisms remain poorly understood [11–15]. Some authors describe the induction of apoptosis by reactive oxygen species (ROS) [13,14]. Other authors explain plasma effects by mechanisms such as necrosis and cell detachment [12]. Furthermore, some studies proved an influence on the cell cycle, like cell cycle arrest or induction of senescence mechanisms by CAP [15,16]. Moreover, our working group showed a restoration of sensitivity in chemo-resistant glioma cells by CAP [17]. The comparison and interpretation of the results of these different CAP investigations regarding “anti-cancer capability” is very difficult. This is also related to the deployment of different technologies (surface micro discharging (SMD), dielectric discharging (DBD), plasma jets or needles) and their applications. Therefore, different chemistry, composition and concentrations of the plasma products, reactive oxygen and nitrogen species, electrons, ions, UV light and temperature were investigated. Nevertheless, almost all studies could see an antitumoral effect on many different malignant cells. Moreover, the effect shows specificity for tumor cells [13–15,18,19].

Underlining this hypothesis our working group showed that CAP treatment of healthy human mucosa tissue cultures (pharyngeal and nasal) induces only a slight cytotoxicity, but no significant genotoxic effects up to 120 seconds of plasma treatment [20].

The aim of this study was the evaluation of the antitumoral efficacy of our CAP device against two squamous head and neck cancer cells lines (OSC-19 and FaDu) and the investigation of the possible mechanisms causing this effect. The methodical focus was set on acute cytotoxicity (MTT-Assay), DNA-fragmentation (Comet Assay) and induction of apoptosis (AnnexinV/PI). Plasma treatment was performed by a hand-held and battery-driven device that operated to the Surface Micro Discharge (SMD) technology [21].

## Material and Methods

### Cell Culture

The FaDu cell line, originating from a low graded tongue cancer [22], was purchased from the American Type Culture Collection (ATCC, Manassas, VA, USA). The OSC 19 cell line obtained 1989 from a hypopharyngeal cancer is growing in vivo as a well differentiated squamous cell carcinoma with a high rate of metastasis [23] and was purchased from JCBR Cell BANK. OSC 19 cells were grown in DMEM/Ham´s F-12 (Biochrom AG, Berlin, Germany) and FaDu cells in DMEM (Biochrom). Both cell lines were also supplemented with 10% fetal bovine serum (Gibco, Carlsbad, CA, USA) and 10 U/ml Penicillin-Streptomycin (Biochrom). FaDu cells were additionally supplemented with 1% non-essential amino acids (Invitrogen, Karlsruhe, Germany) and each 1% of L-Glutamine and Sodium Pyruvate (Biochrom). The cells were maintained at 37°C with 5% CO^2^ under humidified conditions.

### Plasma Treatment

**Fig 1:**
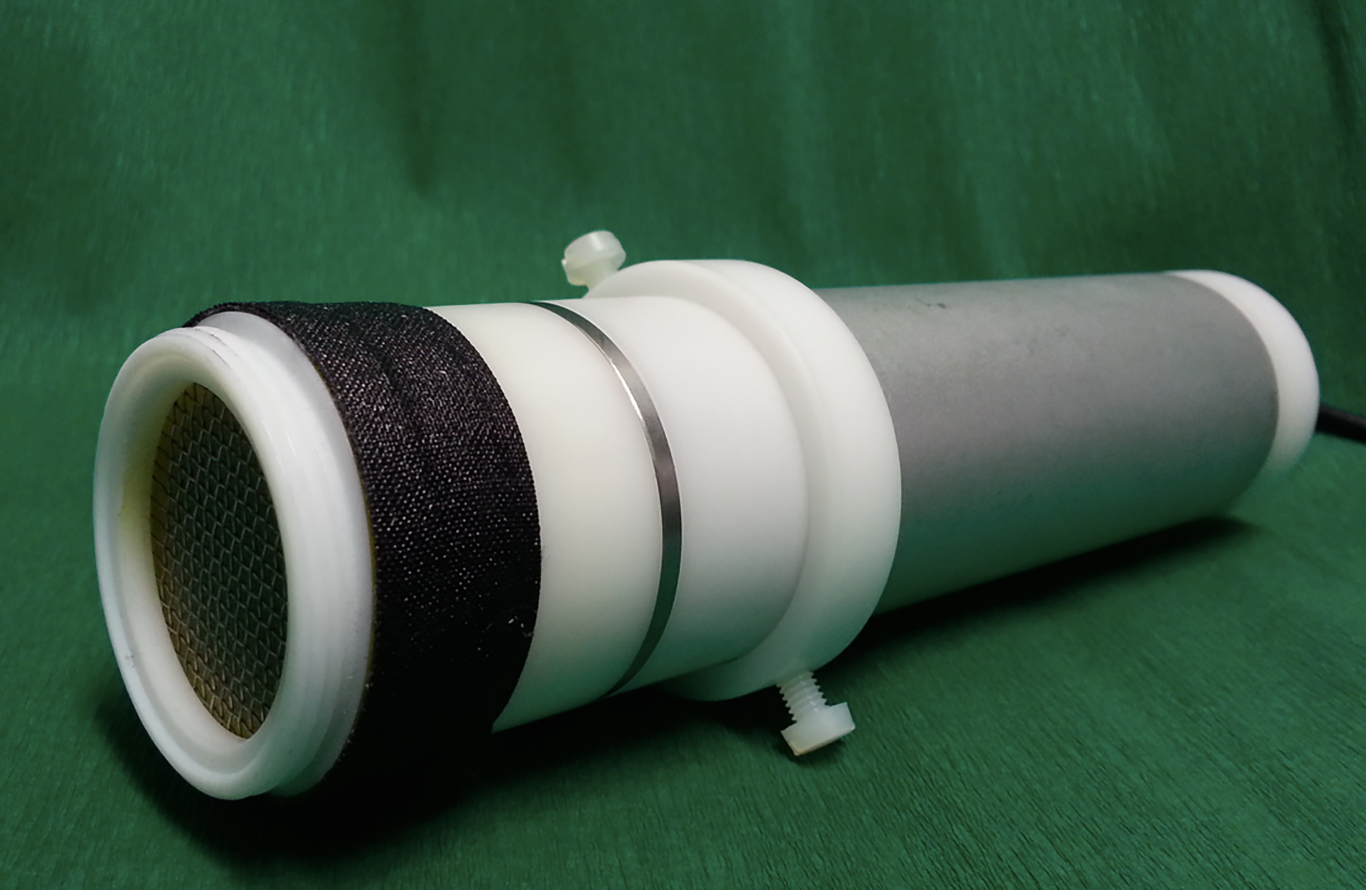
Image of the CAP device–MiniFlatPlaSter®- used in this study.

This study was carried out by the so called MiniFlatPlaSter®, a handheld CAP device which uses the Surface Micro Discharge (SMD) technology for plasma production in air [21] (Fig 1). Details of the device and its plasma chemistry were published in Welz et al. [20] and Maisch et al. [24]. Table 1 shows the main constitutes produced by the plasma device.

**Table 1:** Characteristics of the MiniFlatPlaSter®. The concentration of reactive species was measured as a function of time; the above values are averages over 1 min.

Charged particles	Electrons, ions	At the surface of the SMD electrode ∼10^11^ cm^-3^
Reactive species	O_3_	Mean value for a treatment time of 1 min ∼25 ppm
Reactive species	NO	Mean value for a treatment time of 1 min <1 ppm
Reactive species	NO_2_	Mean value for a treatment time of 1 min <15 ppm
Photons	UV, visible	Power density <0.66 μW cm^−2^
Static electric field		<50 nA cm^−2^
Electrical current through the samples		0032
Heat		At maximum 1°C increase at the 6-well bottom (referring to the ambient temperature for a treatment time of 1 min)

The SMD electrode consists of a glass epoxy board, which is sandwiched by a copper foil layer and a stainless-steel mesh grid. By applying a high pulse-like voltage of 7 kV (peak-to-peak) with a repetition frequency of 6.75 kHz the plasma is produced homogenously on the mesh grid side in the ambient air. Thus, the generated species are transported to the cells by diffusion. In contrast to Dielectric Barrier Discharge (DBD) devices–the used SMD devices produces very low currents (Table 1), which ensures a safe application if applied in vivo.

**Fig 2:**
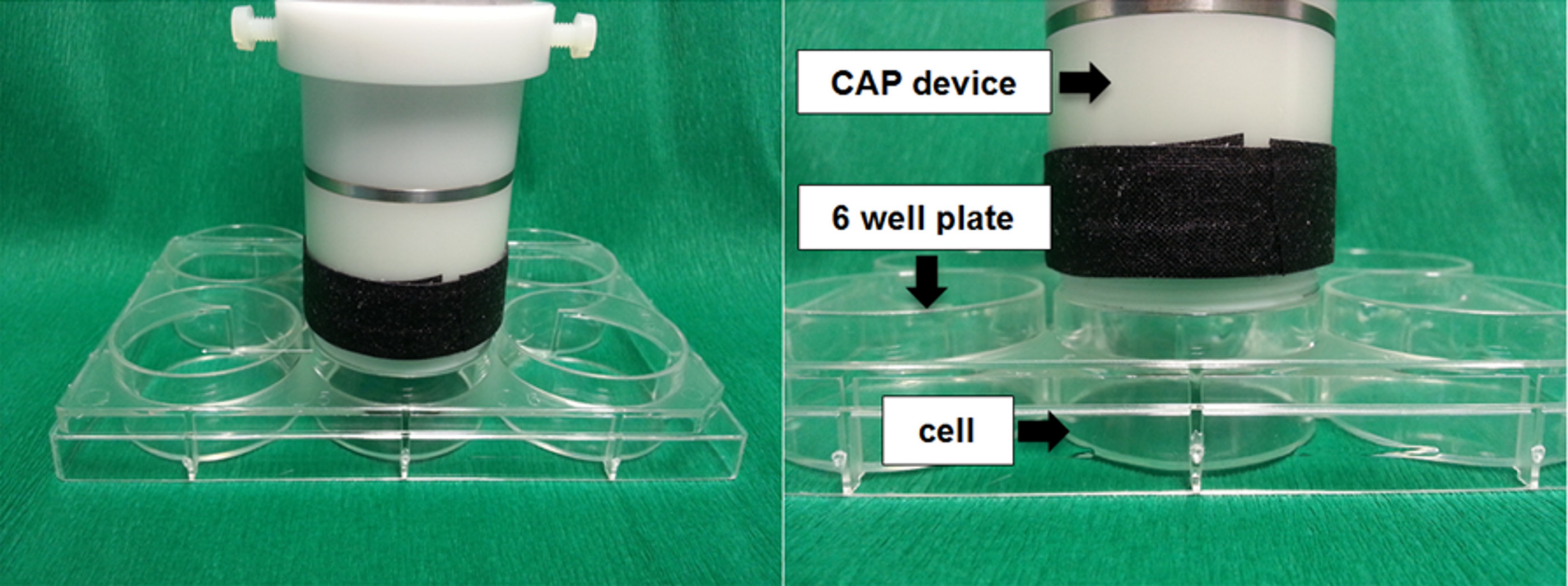
Experimental setup for CAP treatment of the HNSCC cell lines OSC-19 and FaDu.

For experiments 5x10^5^ cancer cells were placed into 6-well plates and were allowed to attach for 24 h hours under same conditions described above. Before the CAP treatment, cell culture medium was removed so that cells weren´t covered by liquid. The CAP electrode was constructed with a diameter of 28 mm, so that it exactly fits the rim of one well. This closed volume condition throughout the CAP treatment confine the produced species (Fig 2) inside. The distance between the electrode and the cells equaled 17.5 ± 0.5 mm. The temperature at the bottom of the well did not increase by more than one degree Celsius after 180 s of CAP treatment. Together with the application by diffusion, dehydration effects on the cells are considered to be unlikely. The CAP treatment times of 30, 60, 90, 120 and 180 s were used. To rule out non-specific plasma effects (such as “drying effects”) as a possible bias, a separate control for each treatment time was carried out. This means that the respective controls were treated in the same way as the corresponding plasma treatment experiments except that the control cells did not receive a plasma treatment. Cell medium was added to all wells immediately after CAP exposure.

### Cell Viability/MTT-Assay

Cytotoxicity measurements were performed by using the Cell Proliferation Kit I of Roche Diagnostics (Roche Diagnostics GmbH, Mannheim, Germany) according to the instruction manual. To monitor cell viability changes after different CAP treatment times, as described above, the treated cells and the respective controls were trypsinized and seeded at 8000 cells/well (100 μl) in a 96 well plate. Cell toxicity was measured 24 h after plasma treatment replacing the culture medium with 10 μl labeling medium containing 0,5 mg/ml MTT. After 4 h of incubation in a humidified atmosphere (37°C with 5% CO_2_), 100 μl MTT-staining solution was added into each well followed by incubation overnight. The purple formazan dye was quantified using a VERSAmax™ ELISA- Reader (Molecular Devices GmbH, Biberach, Germany) at a wavelength of 550 nm. The reference wavelength corresponds to 690 nm. Every experiment contained triplicate measurements of cell viability reduction of each plasma treatment time. Furthermore, each experiment was repeated five times (measurements for each treatment time n = 15). The mean cell viability reduction curves were standardized to the reduced percentage of living cells, whereas the control cells were set at 0%.

### DNA damage/Comet assay

For quantifying DNA damages after the different CAP treatment, assessment of the alkaline microgel electrophoresis (Comet assay) was performed. Basically the protocol is able to detect DNA-strand breaks in single cells, alkali-labile sites (ALSs) and incomplete excision repair [25].

For measurements, trypsinisation was performed directly after CAP treatment by incubation with 1 ml trypsin/EDTA solution for 8–10 min. After neutralization and centrifugation (10 min, 900 U/min), cell counting and cell viability screen by trypan blue exclusion test were performed. On each slide 200.000 cells were applied and covered with 0.5% normal melting agarose (Biozym) to ensure stability to the agarose layers. For cell lysis the slides were covered with alkali solution for 1 h (10% DMSO, 1% Triton-X, 2.5 M NaCl, 10mM Trizma-Base, 100 mM Na_2_ EDTA and 1% N -lauroylsarcosine sodium salt). Then the slides were placed in the gel electrophoresis chamber (Renner, Dannstadt, Germany) and incubated for 20 min with alkaline buffer solution containing 300 mM NaOH and 1mM Na2EDTA at pH 13.2. After this DNA unwinding period, electrophoresis was started at 0.8 V cm−1 and 300 mA and continued for 20 min followed by neutralization (Trisma base, 400mM, pH 7.5; Merck, Germany).

Prior to analysis with a DMLB microscope (Leica, Bensheim, Germany) fluorescent DNA staining was performed with 75 μl ethidium bromide (Sigma; [51 μM]). 80 cell nuclei per slide (2 slides per CAP treatment time) were selected with random pattern and digitized with the attached monochrome CCD camera (Cohu Inc., San Diego, CA, USA).

**Fig 3:**
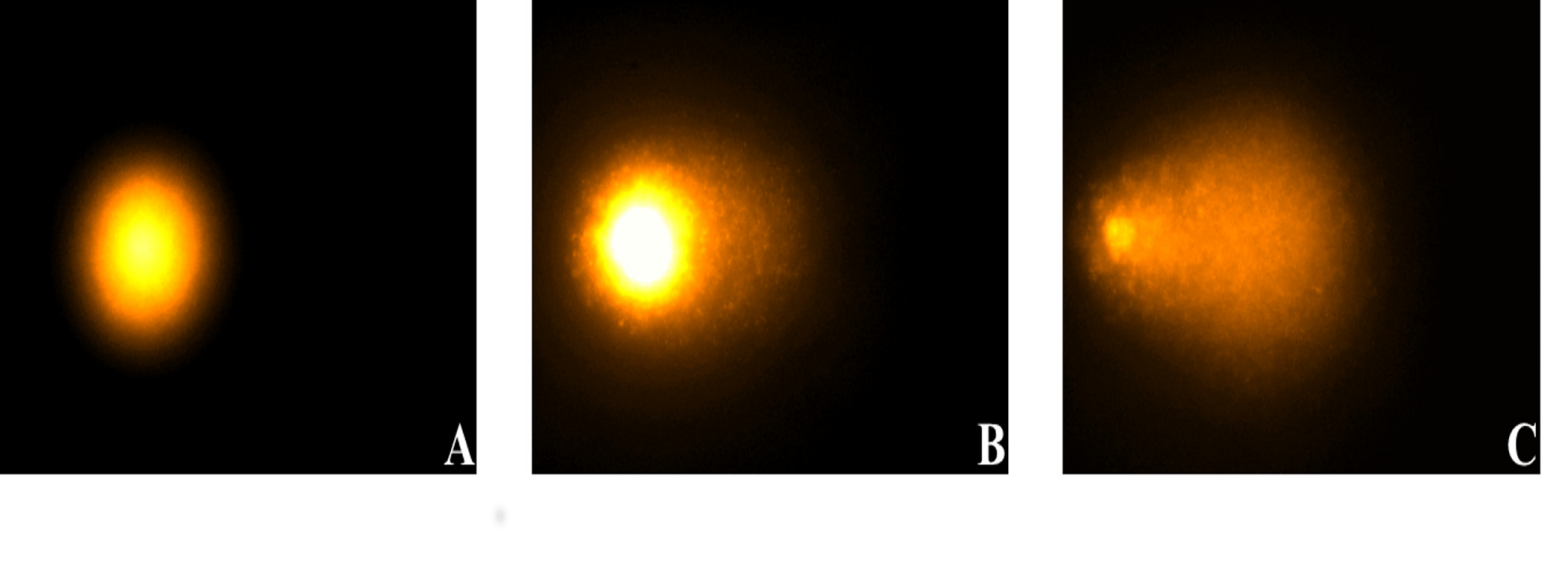
Comet assay images after DNA-staining with ethidium bromide. **A** Undamaged OSC-19 cell with intact DNA and no migration. DNA fragmentation leads to a faster and further migration into the electric field, which results in a figure shaped like a comet with undamaged DNA in the head and damaged DNA in the tail (**B+C**). The brighter and longer the tail, the higher the level of DNA fragmentation. **B** OSC-19 cell with a moderate CAP induced DNA-damage. **C** Representative image of high DNA-fragmentation.

A high DNA fragmentation/DNA-damage leads to a faster and further migration in the electric field, since intact DNA does not migrate (Fig 3). DNA-migration was measured by the image analysis software Komet++ (Kinetic Imaging, Liverpool, UK) using the % of DNA in tail. The % tail DNA is a measure of the relative fluorescent intensity in the head and tail.

### Apoptosis detection

**Fig 4:**
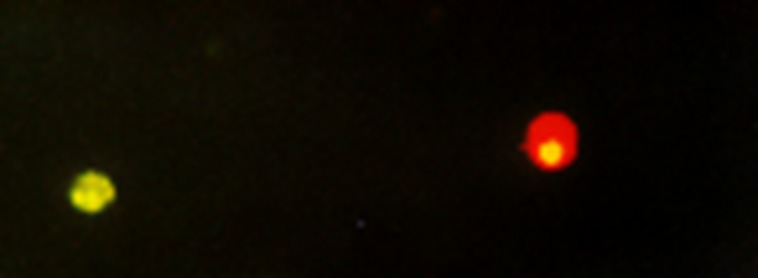
Annexin V/PI Staining of HNSCC cells. Results obtained by fluorescence microscopy show that early apoptotic cells have bound Annexin-FITC to the phosphatidylserin on the membrane surface (green cell). As apoptosis progressed, the plasma membrane integrity gets lost, and the propidium iodide is able to bind to nucleotid DNA, so that late apoptotic or necrotic cells appear in red.

Induction of apoptosis 24 h after the CAP treatment was investigated with fluorescence microscopy using the Annexin V-FITC detection kit (PromoKine, Heidelberg). CAP treatment and trypsinisation were carried out as described above. For experiments 1x10^5^ cells were resuspended in 500 μl binding puffer solution and incubated for 5 min under red light with 5 μl Annexin V-FITC and 5 μl Propidium iodid (PI). Translocation of phosphatidylserin (PS) from the internal to the external face of the plasma membrane is an early step of Apoptosis. Annexin-V is a calcium-dependent phospholipid binding protein with a high affinity to PS and can therefore be used as a sensitive marker for the apoptotic cells. Combining Annexin-V labelling with a PI staining allows differentiation between viable, early apoptotic and late apoptotic/necrotic cells (Fig 4). 100 cells per CAP treatment were counted and early apoptotic cells were identified and indexes were calculated.

### Statistical analysis

Unless elsewhere noted data were reported as the arithmetic mean ± SEM. The SigmaPlot software for Windows Version 12.0 (2011 by Systat Software Inc. CA, USA) was used for statistical analyses. For calculating the statistical significance of the results the Friedman Repeated Measures Analysis of Variance on Ranks and All Pairwise Multiple Comparison Procedures (Student-Newman-Keuls Method) were used. Differences were considered significantly at p-values < 0.05, prior the statistical analysis.

## Results

### Dose dependent reduction of cell viability after CAP treatment

**Fig 5:**
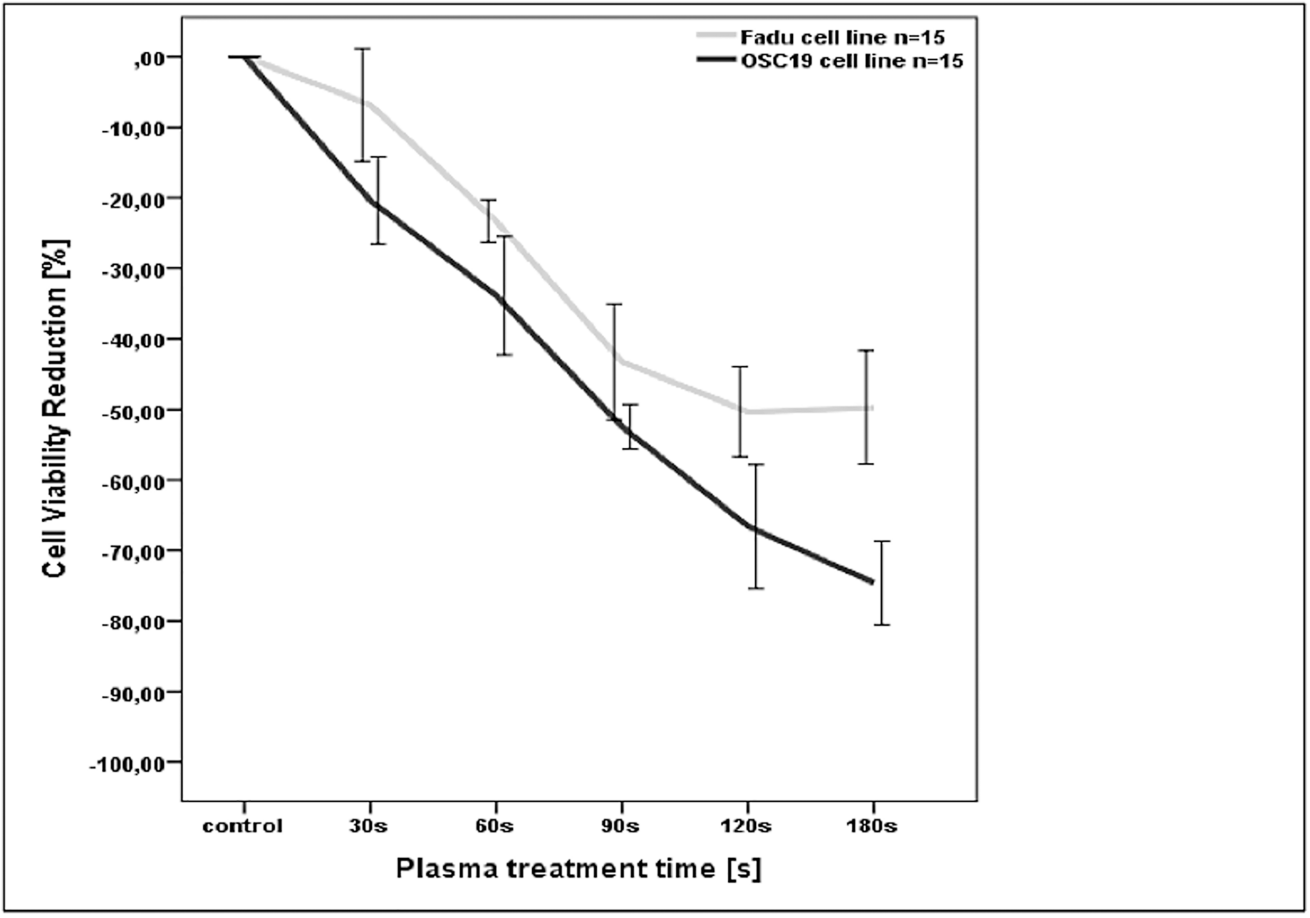
Measured cell viability reduction for different CAP treatment times. For both HNSCC cell lines the viability decreases for increasing CAP treatment times. Standard errors of the mean, depicted by whisker blots.

Fig 5 shows the reduction of the viability of OSC-19 and FaDu cancer cells after CAP exposure for treatments of 30, 60, 90, 120 and 180 s compared to the untreated controls. Cell viability was quantified using the MTT-Assay as described in the methods section. Reduction of viability was set to 0% so that the results -expressed in negative per cent of the control- allow the visual comparison between the two cell lines. For FaDu cell cells viability was significantly reduced by 4,8% (p<0,05) for a CAP treatment time of 30 s as compared with the controls. A treatment time of 60 s induced a reduction of 23,4% (p<0,05) which was statistical significant compared with the controls and 30s. Longer treatment times of 90 s and 120 s dropped viability by 43.6 respectively by 49.2%. In comparison to the controls and the prior shorter treatments times of 30 s and 60 s these reductions were highly significant (p<0,001). A treatment time of 180s decreases FaDu viability by 51,4%. In the case of 90 s and 120 s this was a significant reduction compared to controls and treatment times up to 60 s (p<0,05). The comparison of 90, 120 and 180 s showed no further significant cell viability reduction (p>0,05).

For OSC 19 cells viability was reduced by 20% after a treatment time of 30 s and by 31,2% for 60 s respectively. Longer treatment times (90 s and 120 s) induced further reduction of cell viability by 51,7% (90 s) and 64,1%(120 s). All viability reductions were statistically significant compared to the untreated controls and to the prior shorter treatment times (p<0,05). 180 s of treatment lead to a further reduction by 73,3%, which was significant compared to the controls and to treatment times up to 90 s (p<0,05).

### Induction of DNA-damage by CAP

**Fig 6:**
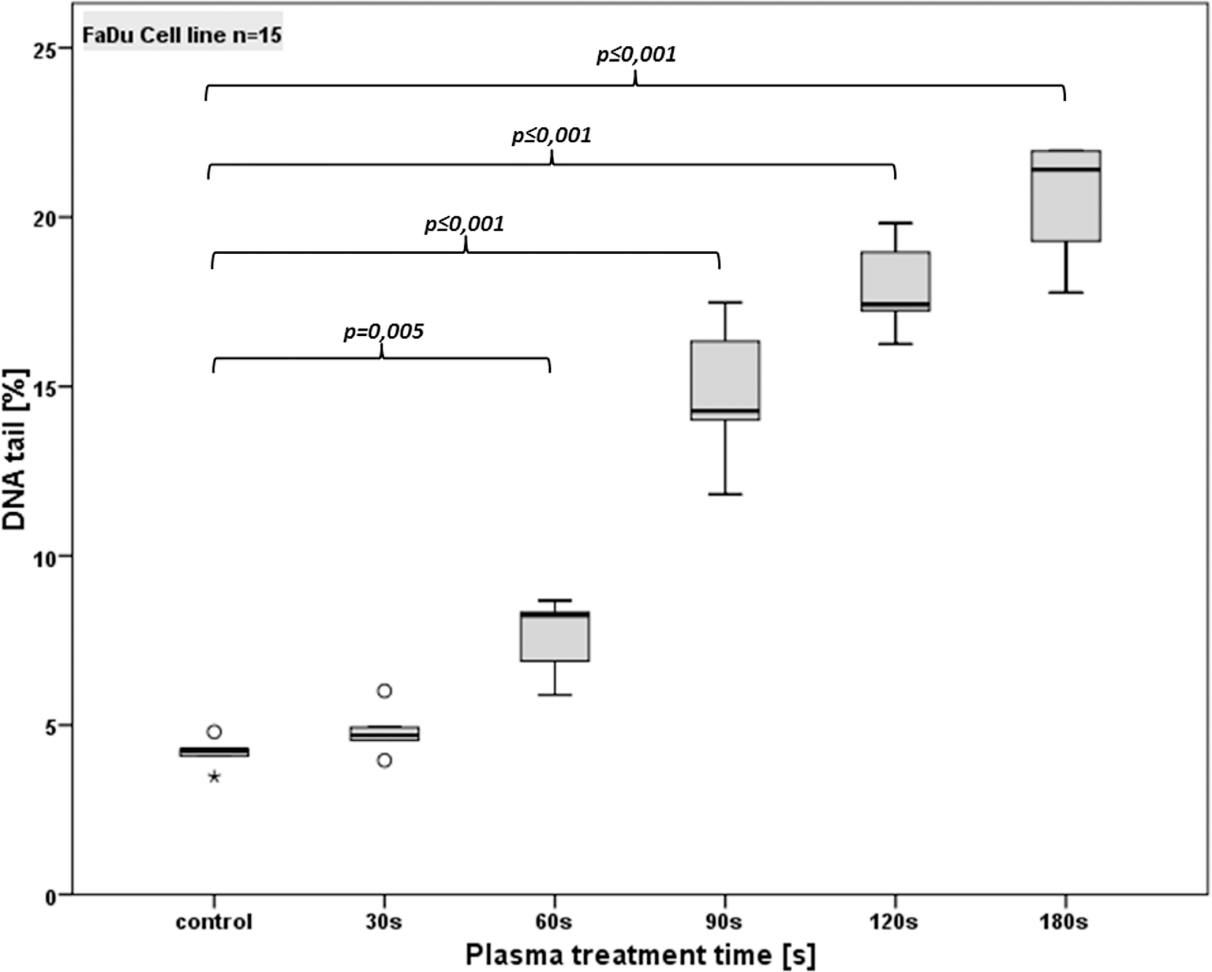
Detection of DNA damage (using the comet assay) in FaDu cells after different CAP treatment times. Standard box-plots (lower quartile, median, upper quartile) were used to illustrate the results. Dots denote mild statistical outliers (between 1.5 and 3 times interquartile range (IQR)); asterisks denote extreme statistical outliers (more than 3 times IQR).

**Fig 7:**
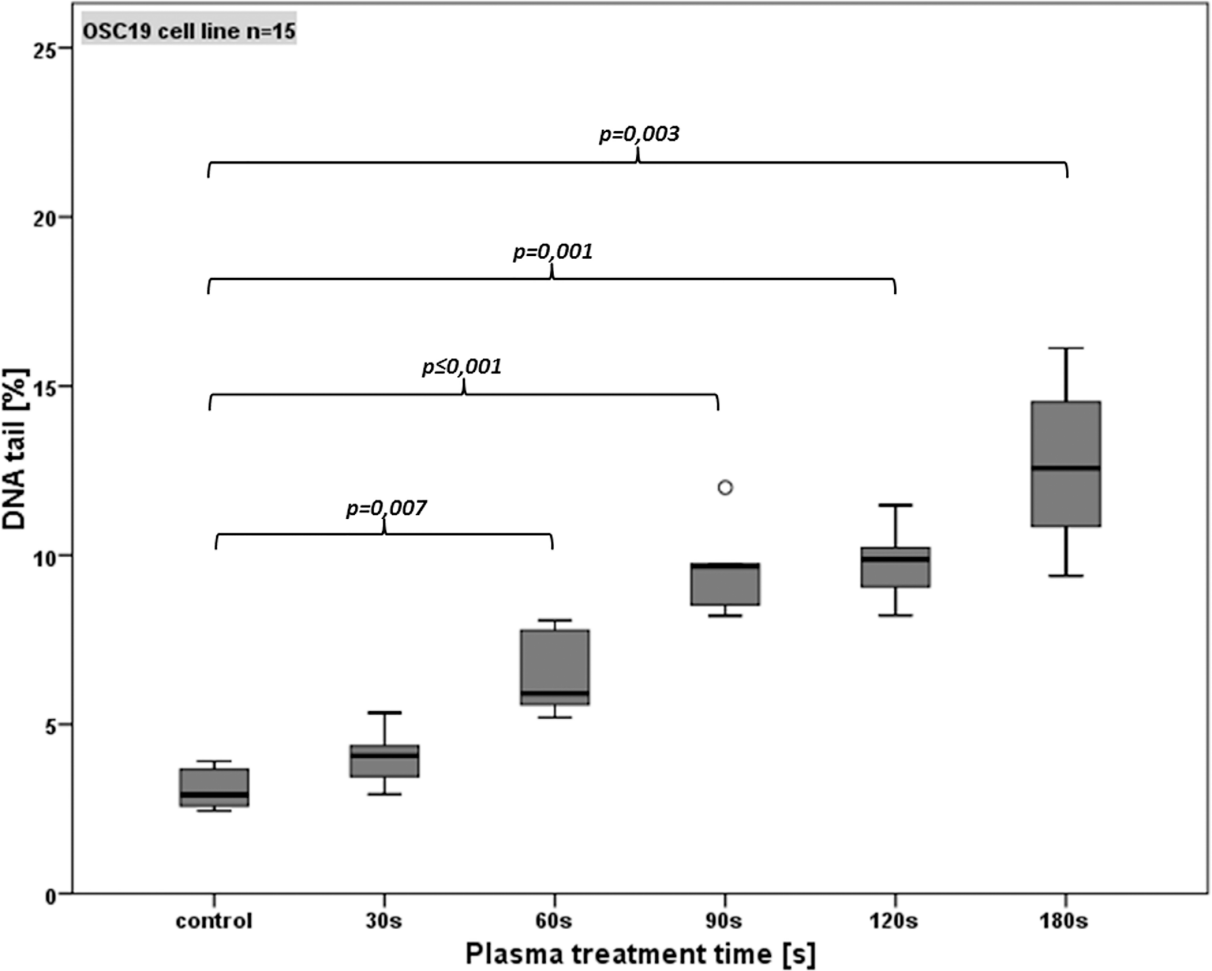
Detection of DNA damage (using the comet assay) in OSC-19 cells after different CAP treatment times. Standard box-plots (lower quartile, median, upper quartile) were used to illustrate the results. Dots denote mild statistical outliers (between 1.5 and 3 times interquartile range (IQR)).

To investigate if the time dependent reduction in cell viability and the increasing rate of apoptosis is caused by CAP induced DNA damage the alkaline microgel electrophoresis assay was performed. Figs 6 and 7 show the percentage of tail-DNA of each cell line in detail.

Analysis of experiments using the FaDu cell line (Fig 6) revealed no significant increase of DNA damage compared with the controls (4.2%; p > 0.05) for 30 s of plasma treatment (4.8% of DNA in tail). For 60 s a significant increase of % tail DNA (7.6%) compared with the untreated controls (p<0.05) and CAP duration of 30 s (p < 0.05) was observed. Longer durations increased the DNA fragmentation to 14.8% (90 s) respectively to 17.9% (120 s). Compared to the controls, 30 s and 60 s of CAP treatment this increment was statistically significant (p<0.05). The increasing DNA damage from 90 s to 120 s of CAP treatment showed a statistically significance as well. CAP duration of 180 s lead to a further DNA fragmentation of 20.5% DNA in the tail. Compared with the untreated controls and all other treatment times this increment of 180 s was statistically significant (p<0.05).

For OSC 19 cells 30 s plasma treatment induced a DNA fragmentation of 4.0%, which was not significant as compared to the untreated controls (3.1%; p>0.05). Longer CAP treatment (60 s and 90 s) induced an increase of DNA damage to 6.5% (60s) and 9.6% (90 s). Similar to FaDu cell line experiments this increase showed a significance (p<0.05) compared to the controls and each prior treatment time. Indeed, for 120 s and 180 s a further growth of DNA fragmentation was observed (9.8% for 120 s, 12.7% for 180 s) but this increment was not significant compared to the prior duration of 90 s (p>0.05). Fig 7 shows the % tail DNA for OSC 19 cells in detail.

### CAP induced apoptosis

**Fig 8:**
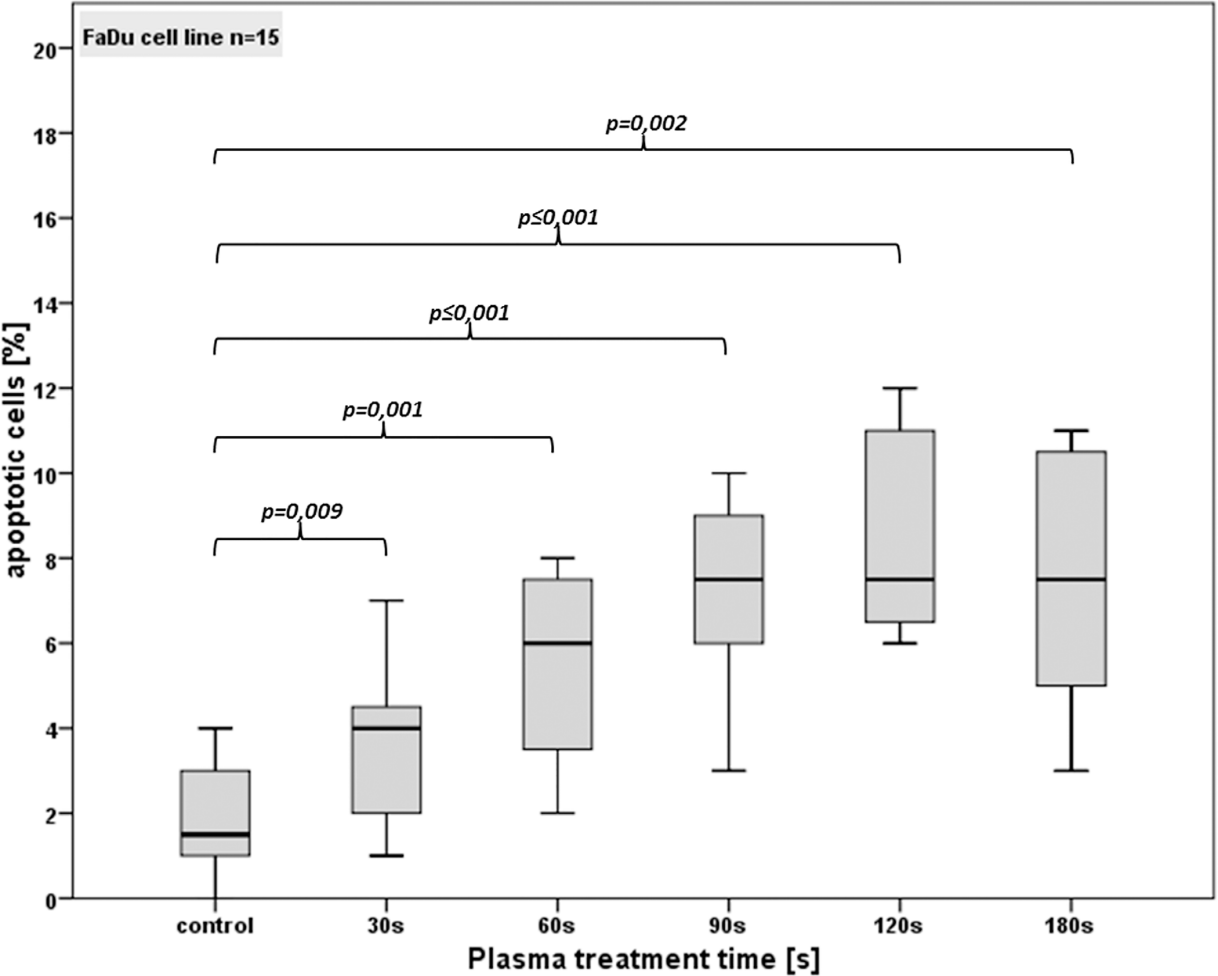
Standard box-plot of apoptotic cells for FaDu cell line after different CAP treatment times.

**Fig 9:**
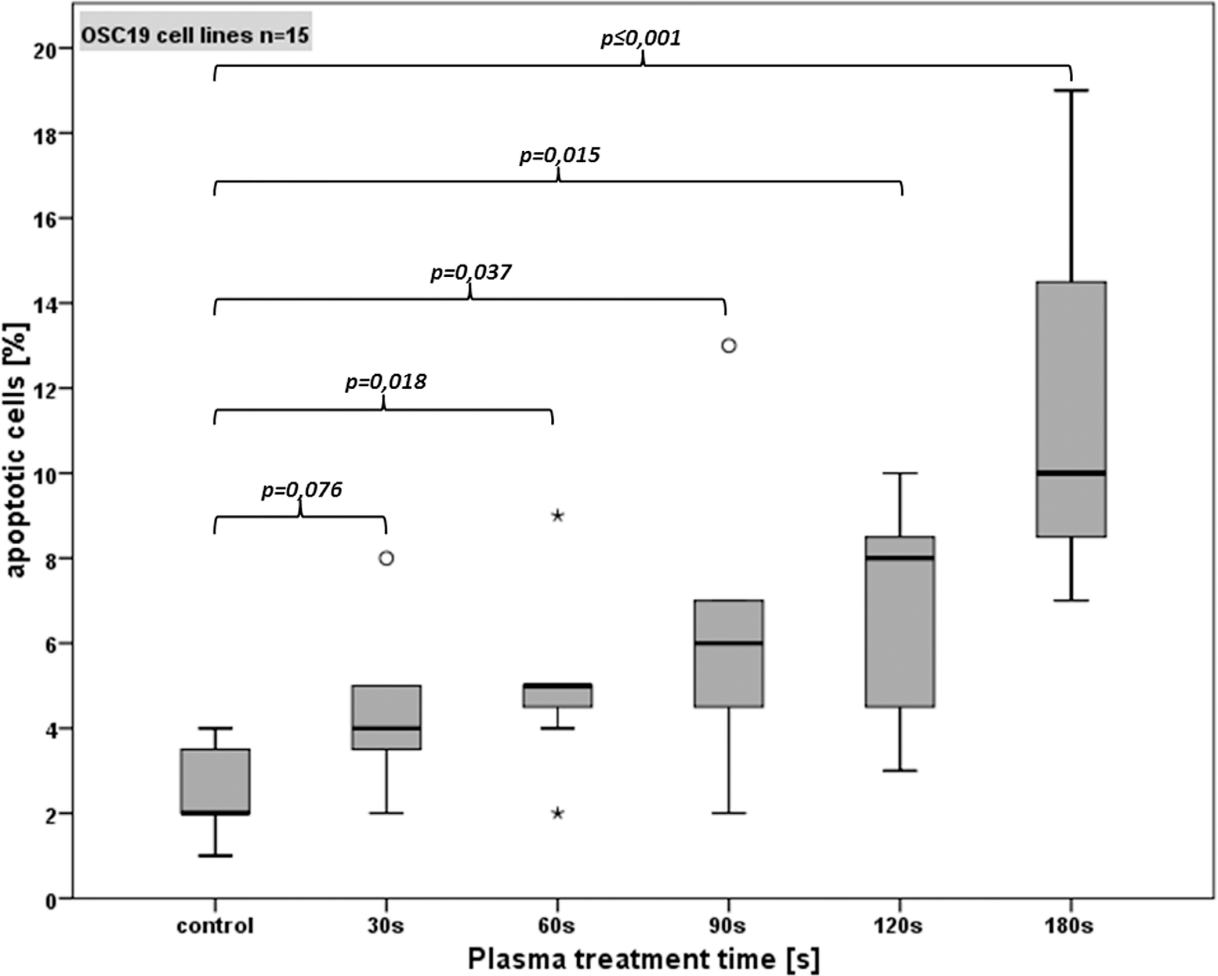
Apoptotic OSC-19 cells after different CAP treatment times.

To clarify if the time dependent increase of DNA damage induces apoptosis and if reduction in cell viability can be explained by programmed cell death, AnnexinV/PI staining was performed 24 h after CAP treatment. As presented in Figs 8 and 9, plasma treatment induced apoptosis in both cell lines. 180 s of plasma treatment resulted in an over 4-fold increase of early apoptotic cells as compared to the controls (p ≤ 0.002). In detail, for FaDu cell line (Fig 8) the untreated control showed an average of 1.9% apoptotic cells. After a CAP treatment time of 30 s a slight increase of apoptosis (3.6%) was detected, which was significant to the controls (p<0.01). After 60 s an average of 5.5% of the cells were apoptotic and a duration of 90 s increased the rate of apoptotic cells to 7.3%. Compared to the controls and to each shorter treatment time this increase was statistically significant (p<0.05). Longer treatment times of 120 s and 180 s showed increasing rates of apoptotic cells of 8.5%, respectively 7.5%. Compared to the controls and to treatment times up to 60 s these rates were statistically significant (p<0.05), but no significance was seen as compared with one another and to 90 s (p>0.05).

The untreated OSC 19 cells showed an average of 2.6% of apoptotic cells (Fig 9). For CAP treatment times of 30 s, a slight increase of apoptosis to 4.4% was detected, but these data are not significant. A CAP treatment time of 60 s showed an average of 5.5% apoptotic cells, which is a significant increase compared with the controls (p < 0.05). The 90 s treatment increased the rate of apoptotic cells to 5.9% and 120 s to 6.4%. Both durations showed a significant increase of apoptotic cell rate compared to untreated cell (p<0,05), but no significance was seen when comparing it to shorter treatment times. A treatment time of 180 s induced 11.3% apoptotic cells. This increment was statistically significant compared with the control and all prior treatment times (p < 0.05).

## Discussion

With being one of the most potential agents against any microorganism and the proof of its efficacy in clinical trials, CAP medicine is a rapidly growing research topic and develops increased importance in health care. Cancer treatment is the most promising new medical application of CAP. In vitro and in vivo studies on different kinds of tumours revealed strong and specific effects on cancer cells and inhibition of cancer growth [14–16,18,19]. It seems, that CAP can induce cell cycle arrest and at sufficiently high doses cause apoptosis and necrosis in cancer cells. However, the exact mechanisms of CAP effects remain unclear.

The production of reactive nitrogen and oxygen species by CAP is thought to be a key player initiating the observed direct antitumoral effects. Several publications suggest an increase of intracellular ROS/RNS levels upon CAP treatment, thereby inducing DNA damage and apoptosis in tumor cells [13,14,26]. Vandamme et al. showed a dose depending induction of apoptosis after CAP treatment of malignant glioma (U87MG) and colorectal carcinoma (HCT-116) cells [14]. The apoptotic cell death was also explained as a result of ROS effects. Besides ROS induced DNA-damage followed by apoptosis there are data describing an acute cytotoxic/necrotic effect [12]. As of today there are no data showing efficacy of a SMD plasma device generated CAP against head and neck cancer cells. Nevertheless, especially for organs with natural orifice like skin and mucosa of the upper aerodigestive tract (oral cavity, oropharynx, parts of the hypopharynx and larynx) CAP unveils highly potent treatment options for cancer of the head and neck. Moreover our handheld SMD device, generating plasma in the surrounding air can reach HNSCC via diffusion while avoiding a carrier gas or gas flow. Thus, we hypothesize that our device can induce a treatment time depending cell death in HNSCC cell lines.

The cell viability assay showed that CAP had a significant anti-tumor activity on FaDu and OSC-19 cell lines with an IC_50_ of 120 sec (FaDu) and 90 sec (OSC-19) exposure time. In OSC-19 cell lines, duration of 180s led to about 75% cell death. These results are in line with several studies, which showed an anti-tumor activity of CAP on various malignant cell lines including melanoma, glioma, hepatocellular, and colorectal carcinoma [11,13,14,27–30]. In comparison, our investigations on healthy mucosal cell cultures with the same device and under the same experimental conditions showed only a cell viability reduction of 15% after 120 s of plasma treatment [20]. Consequently we proved that our SMD plasma device is effective against HNSCC cell lines while healthy cells stay nearly unaffected.

Besides surgery, radiotherapy (RT) is the major modality in primary HNSCC treatment and indispensable for adjuvant strategies [31]. The main mechanism for anticancer activity respectively capability of radiotherapy are radiation induced DNA damages which initiate cell cycle changes and signal cascades finally leading to cell death. To evaluate if CAP is also able to induce DNA damage in HNSCC cells which could be responsible for a treatment time dependent viability reduction, the alkaline microgel electrophoresis technique (Comet assay) was performed being very sensitive for detecting DNA single-strand breaks (SSBs), double strand breaks (DSBs) and alkali-labile sites (ALSs). Because SSB and/or ALS occur more likely than DSB and other DNA alterations such as DNA crosslinks or intercalations, the alkaline mircogel electrophoresis is highly sensitive for quantifying low levels of DNA damage, which makes it superior to other genotoxicity tests [25,32]. Despite this high sensitivity it has to be mentioned that DNA-repair mechanisms and their consequence on the cell viability, are not considered by this method Our test observed a significant treatment time, respectively dose dependent increase of DNA-damage in both HNSCC cell lines. Compared to the exposure of healthy mucosal tissue cultures to CAP [20], an up to four fold DNA fragmentation in cancer cells was detected. Hence our results show that CAP or its products interact with the DNA of HNSCC cell lines, and is able to induce lethal DNA damage, which can explain the dose dependent anticancer activity of our SMD plasma device.

For evaluation if dose dependent DNA damage induces apoptosis and whether the treatment time dependent reduction of cell viability is a result of an apoptotic process, we performed the AnnexinV/PI staining. After 60 s treatment time and longer, our CAP device induced apoptotic cell death in both cell lines as compared to untreated controls. Nevertheless comparing the treatment times separately, there was no significant dose dependent increase of apoptotic cells. Only long treatment times correspondingly high plasma doses induced a high rate of apoptotic cells in the OSC-19 cell line which is in accordance to the results of Arndt et al. In that study, which was carried out with the same plasma device we used (MiniFlatPlaSter®), and therefore had the same plasma physics and chemical compositions, treatment times of 120 s induces DNA damage and strongly increased apoptosis in melanoma cell lines. In case of shorter treatment times almost no apoptotic cell rate was observed but revealed a senescence induction [16].

We again would emphasize that a comparison of various experiments carried out with different CAP devices is difficult due to different CAP parameters and designs i.e. DBD technology, SMD technology, power input, voltage, frequency, carrier gas etc. resulting in relevant differences in the production of their components. But experiments carried out with the equal plasma device also lead to diverging results when different cell lines (malignant/non-malignant) are targeted. Nevertheless a selective cytotoxic effect on cancer cells is emerging in correlation to the results of various experiments of CAP on malignant cells [14,15,18,19]. The mechanism for this selectivity remains unclear. But in our investigations on epithelial cells, CAP seems to have similarities to the anticancer mechanisms of radiation, with showing a high DNA damage and cell death in high proliferating cancer cells, while low proliferating healthy mucosal tissue seems to be less affected by the same treatment. Nevertheless to claim selectivity regarding HNSCC cells and healthy tissue, more comparable investigations on cancer cells of the head and neck and mucosal tissue have to be carried out [20].This is the first study, evaluating the effects of an SMD device generated CAP on HNSCC cell lines. A dose dependent high reduction of cell viability was observed which is partially caused by apoptosis induction. Moreover a treatment time depending high DNA fragmentation was detected, which is probably the key player concerning cancer cell death. With these results we confirmed that SMD plasma technology is a highly promising new approach in head and neck cancer treatment.

## Supporting Information

S1 File Raw Data. (XLSX)

S2 File Statistic report. (DOCX)
