# Supplementary material for: Cold Atmospheric Plasma: A Promising Complementary Therapy for Squamous Head and Neck Cancer
Source: PLoS One. 2015 Nov 20;10(11):e0141827. doi: 10.1371/journal.pone.0141827 (PMC4654510; doi:10.1371/journal.pone.0141827)
Supplement: S2 File — (DOCX) [file pone.0141827.s002.docx]

**Methode**

SigmaPlot for Windows Version 12.0

2011 by Systat Software Inc.

Friedman Repeated Measures Analysis of Variance on Ranks, All Pairwise Multiple Comparison Procedures (Student-Newman-Keuls Method)

**FaDu cells**

delta Vitalität

The differences in the median values among the treatment groups are greater than would be expected by chance; there is a statistically significant difference (P = <0,001)

To isolate the group or groups that differ from the others use a multiple comparison procedure:

Comparison Diff of Ranks q P<0,05

**0 vs 120 57,000 7,867 Yes**

**0 vs 180 53,000 8,655 Yes**

**0 vs 90 51,000 10,200 Yes**

**0 vs 60 25,000 6,455 Yes**

**0 vs 30 9,000 3,286 Yes**

**30 vs 120 48,000 7,838 Yes**

**30 vs 180 44,000 8,800 Yes**

**30 vs 90 42,000 10,844 Yes**

**30 vs 60 16,000 5,842 Yes**

**60 vs 120 32,000 6,400 Yes**

**60 vs 180 28,000 7,230 Yes**

**60 vs 90 26,000 9,494 Yes**

90 vs 120 6,000 1,549 No

90 vs 180 2,000 0,730 Do Not Test

180 vs 120 4,000 1,461 Do Not Test

% DNA tail

The differences in the median values among the treatment groups are greater than would be expected by chance; there is a statistically significant difference (P = <0,001)

To isolate the group or groups that differ from the others use a multiple comparison procedure:

Comparison Diff of Ranks q P<0,05

**180 vs 0 24,000 5,737 Yes**

**180 vs 30 21,000 5,940 Yes**

**180 vs 60 15,000 5,196 Yes**

**180 vs 90 10,000 4,472 Yes**

**180 vs 120 5,000 3,162 Yes**

**120 vs 0 19,000 5,374 Yes**

**120 vs 30 16,000 5,543 Yes**

**120 vs 60 10,000 4,472 Yes**

**120 vs 90 5,000 3,162 Yes**

**90 vs 0 14,000 4,850 Yes**

**90 vs 30 11,000 4,919 Yes**

**90 vs 60 5,000 3,162 Yes**

**60 vs 0 9,000 4,025 Yes**

**60 vs 30 6,000 3,795 Yes**

30 vs 0 3,000 1,897 No

Apoptose %

The differences in the median values among the treatment groups are not great enough to exclude the possibility that the difference is due to random sampling variability; there is not a statistically significant difference (P = 0,416)

**FaDu cells**

delta Vitalität

The differences in the median values among the treatment groups are greater than would be expected by chance; there is a statistically significant difference (P = <0,001)

To isolate the group or groups that differ from the others use a multiple comparison procedure:

Comparison Diff of Ranks q P<0,05

**0 vs 180 69,000 9,523 Yes**

**0 vs 120 63,000 10,288 Yes**

**0 vs 90 47,000 9,400 Yes**

**0 vs 60 27,000 6,971 Yes**

**0 vs 30 19,000 6,938 Yes**

**30 vs 180 50,000 8,165 Yes**

**30 vs 120 44,000 8,800 Yes**

**30 vs 90 28,000 7,230 Yes**

**30 vs 60 8,000 2,921 Yes**

**60 vs 180 42,000 8,400 Yes**

**60 vs 120 36,000 9,295 Yes**

**60 vs 90 20,000 7,303 Yes**

**90 vs 180 22,000 5,680 Yes**

**90 vs 120 16,000 5,842 Yes**

120 vs 180 6,000 2,191 No

% DNA tail

The differences in the median values among the treatment groups are greater than would be expected by chance; there is a statistically significant difference (P = <0,001)

To isolate the group or groups that differ from the others use a multiple comparison procedure:

Comparison Diff of Ranks q P<0,05

**180 vs 0 22,000 5,259 Yes**

**180 vs 30 21,000 5,940 Yes**

**180 vs 60 14,000 4,850 Yes**

180 vs 120 6,000 2,683 No

180 vs 90 6,000 3,795 Do Not Test

**90 vs 0 16,000 4,525 Yes**

**90 vs 30 15,000 5,196 Yes**

**90 vs 60 8,000 3,578 Yes**

90 vs 120 0,000 0,000 Do Not Test

**120 vs 0 16,000 5,543 Yes**

**120 vs 30 15,000 6,708 Yes**

**120 vs 60 8,000 5,060 Yes**

**60 vs 0 8,000 3,578 Yes**

**60 vs 30 7,000 4,427 Yes**

30 vs 0 1,000 0,632 No

Apoptose %

The differences in the median values among the treatment groups are not great enough to exclude the possibility that the difference is due to random sampling variability; there is not a statistically significant difference (P = 0,416)
